# A machine learning–based prediction model for delirium risk in malnourished elderly ICU patients with SHAP interpretability

**DOI:** 10.3389/fnut.2026.1781117

**Published:** 2026-07-20

**Authors:** Chunmei Zhang, Zhiyi Xie, Fengzhen Chen, Qitian Zhang

**Affiliations:** 1 Department of Cardiology, Zhangzhou Affiliated Hospital of Fujian Medical University, Zhangzhou, Fujian, China; 2 Department of Internal Medicine II, Zhangpu County Traditional Chinese Medicine Hospital, Zhangzhou, Fujian, China

**Keywords:** delirium, elderly patients, GNRI, machine learning, malnutrition, SHAP

## Abstract

**Objective:**

To identify risk factors associated with in-hospital delirium among malnourished elderly patients in the intensive care unit (ICU) and to develop and validate a machine learning–based prediction model for early risk stratification.

**Methods:**

Using data from a large single-center ICU database (MIMIC-IV) and a multicenter ICU database (eICU-CRD), elderly patients with malnutrition who met predefined inclusion criteria were enrolled. Multiple machine learning models were developed and systematically compared. Model performance was assessed using the area under the receiver operating characteristic curve, calibration curves, decision curve analysis, precision–recall curves, and additional performance metrics. External validation was conducted in an independent cohort to evaluate model generalizability. The final model was further interpreted using SHapley Additive exPlanations (SHAP), and a corresponding prediction tool was constructed.

**Results:**

In total, 6,449 malnourished elderly ICU patients were included. Patients who developed delirium showed significantly higher disease severity, greater physiological instability, and worse clinical outcomes than those without delirium. Among the evaluated models, the eXtreme Gradient Boosting (XGBoost) model achieved the best overall performance in terms of discrimination, calibration, and net clinical benefit, and demonstrated stable predictive ability in the external validation cohort. The final model included seven predictors: Sequential Organ Failure Assessment (SOFA) score, Glasgow Coma Scale (GCS) score, body temperature, peripheral oxygen saturation (SpO_2_), Geriatric Nutritional Risk Index (GNRI), pH value, and mechanical ventilation. SHAP analysis indicated that disease severity, nutritional risk, and respiratory function–related factors were key contributors to delirium risk.

**Conclusion:**

This study developed and externally validated a machine learning–based prediction model for delirium risk in malnourished elderly ICU patients, with good predictive performance and interpretability. The model may aid in early identification of high-risk individuals and support targeted prevention and individualized management of delirium in clinical settings.

## 1 Introduction

Malnutrition is defined as a state resulting from inadequate nutrient intake or utilization, leading to loss of body weight and muscle mass accompanied by functional impairment, and is clinically manifested by weight loss, fatigue, and reduced physical performance (1). Among elderly patients in the intensive care unit (ICU), malnutrition is highly prevalent and has been closely associated with increased rates of infection and complications, prolonged duration of mechanical ventilation, and higher ICU and 28-day mortality (2, 3). Moreover, malnutrition contributes to extended hospital and ICU length of stay, increased readmission rates, and greater healthcare expenditures, thereby substantially worsening patient prognosis and imposing a considerable burden on healthcare systems (4). Growing evidence suggests that malnutrition is significantly associated with delirium in elderly ICU patients, with delirium risk increasing in parallel with greater severity of malnutrition or higher nutritional risk scores (5). Potential mechanisms include inflammatory and metabolic dysregulation, electrolyte and vitamin deficiencies, impaired immune function, and pharmacokinetic alterations related to hypoalbuminemia and sarcopenia, which collectively disrupt central nervous system function and predispose patients to delirium (5, 6). Once delirium develops in malnourished elderly patients, it is associated with a markedly increased risk of mortality, prolonged hospitalization, and deterioration in long-term cognitive and functional outcomes (7). Therefore, early recognition of delirium and timely intervention targeting reversible risk factors are of critical clinical importance for improving both short- and long-term outcomes in critically ill elderly populations (8).

Existing evidence indicates that malnutrition and various nutritional risk indicators are closely associated with the occurrence of delirium in elderly patients and demonstrate predictive value in ICU and postoperative populations (9, 10). Although delirium prediction models for older adults have been extensively developed and validated across multiple clinical settings, most existing models predominantly focus on organ dysfunction, inflammatory status, and treatment-related factors, while the systematic incorporation of malnutrition—an important and potentially modifiable risk factor—remains limited (11, 12). With the growing recognition of the strengths of machine learning in handling complex clinical data, enhancing predictive accuracy, and supporting individualized decision-making, these approaches have been increasingly applied to delirium risk assessment in the ICU and have shown favorable performance in high-risk populations such as patients with respiratory failure and sepsis (12–14). However, delirium prediction studies specifically targeting malnourished elderly patients, a particularly vulnerable and underexplored population, remain scarce, and the robustness and generalizability of existing models warrant further investigation.

Therefore, using data from multicenter intensive care unit databases, this study systematically integrated demographic characteristics, clinical variables, and treatment-related information of malnourished elderly ICU patients to identify key risk factors for delirium and to develop and validate a delirium risk prediction model. The study aims to provide objective support for early identification of high-risk patients and implementation of individualized intervention strategies, ultimately improving outcomes in malnourished elderly patients and optimizing the utilization of ICU healthcare resources.

## 2 Methods

### 2.1 Data sources and ethics

This study was conducted using two publicly available ICU databases: a large single-center database [MIMIC-IV, version 3.1 (PhysioNet, MIT Laboratory for Computational Physiology, Cambridge, MA, USA; Beth Israel Deaconess Medical Center, Boston, MA, USA)] and a multicenter database [eICU-CRD, version 2.0 (PhysioNet, MIT Laboratory for Computational Physiology, Cambridge, MA, USA; Philips Healthcare, Andover, MA, USA)]. The MIMIC-IV database was used for model development and internal validation, while the eICU-CRD database was employed for external validation. MIMIC-IV was jointly developed by the Massachusetts Institute of Technology and Beth Israel Deaconess Medical Center and contains high-quality, de-identified clinical data from more than 65,000 ICU patients (15). The eICU-CRD is a multicenter database comprising de-identified health information from over 200,000 ICU admissions across more than 200 hospitals in the United States between 2014 and 2015 (16). Both databases were approved by the corresponding institutional review boards and granted waivers of informed consent, with all protected health information rigorously anonymized. The authors completed the Collaborative Institutional Training Initiative (CITI) program (Record ID: 73212477) and signed the required data use agreements. This study was reported in accordance with the Transparent Reporting of a Multivariable Prediction Model for Individual Prognosis or Diagnosis (TRIPOD) statement (17).

### 2.2 Study population

Elderly malnourished patients admitted to the ICU were included in this study. The inclusion criteria were as follows: (a) age ≥ 65 years at ICU admission; (b) malnutrition defined as a Geriatric Nutritional Risk Index (GNRI) < 98 calculated within 24 h after ICU admission. GNRI was calculated using the formula GNRI = 1.489 × albumin (g/L) + 41.7 × (body weight/ideal body weight), with the ratio of body weight to ideal body weight set to 1 when body weight exceeded ideal body weight. Ideal body weight was calculated using the Lorentz formula (men: WLo = H – 100 – (H – 150)/4; women: WLo = H – 100 – (H – 150)/2.5, where H represents height in centimeters) (18). (c) For patients with multiple ICU admissions, only the first ICU admission during the first hospitalization was included. The exclusion criteria were as follows: (a) ICU length of stay less than 1 day; (b) absence of delirium assessment records.

### 2.3 Data collection and definitions

Data extraction was performed using Navicat Premium 17.0 and structured query language (SQL). The following variables were collected: (1) demographic data: age, sex, height, and weight; (2) vital signs: body temperature, heart rate, respiratory rate, and blood pressure; (3) laboratory parameters: complete blood count, blood biochemistry, coagulation profile, blood gas analysis, and lipid profile; (4) comorbidities: acute myocardial infarction, atrial fibrillation, hypertension, diabetes mellitus, hyperlipidemia, chronic obstructive pulmonary disease, pneumonia, chronic kidney disease, and liver cirrhosis; (5) infusion data: vasoactive drug use; (6) other variables: mechanical ventilation, 24-h fluid balance after admission, 28-day mortality, Sequential Organ Failure Assessment (SOFA) score, and Glasgow Coma Scale (GCS) score; (7) outcome variable: occurrence of delirium during hospitalization. All clinical variables, including laboratory parameters, vital signs, and clinical scores (SOFA and GCS), were extracted from the first 24 h after ICU admission and were used to represent baseline clinical status prior to the occurrence of delirium. For variables with multiple measurements within the first 24 h, mean values were calculated and used for analysis to reflect the overall physiological status during the early ICU period. All predictor variables were obtained from the first 24 h after ICU admission to represent baseline clinical status, thereby reducing potential bias related to ICU length of stay.

The primary outcome of this study was the occurrence of delirium during hospitalization. Delirium was identified based on structured records of standardized assessment tools, including the Confusion Assessment Method for the ICU (CAM-ICU) and the Intensive Care Delirium Screening Checklist (ICDSC) (19). Delirium was defined as the presence of at least one positive CAM-ICU assessment or an ICDSC score ≥ 4 during ICU stay. Diagnosis codes and nursing documentation were additionally reviewed to enhance the reliability of delirium ascertainment. Due to the retrospective nature of the databases, detailed information on assessment timing, frequency, and assessor characteristics was not uniformly available.

### 2.4 Data preprocessing and feature selection

During data preprocessing, continuous variables were first screened for outliers using the three-times interquartile range (IQR) method, and suspicious values were manually reviewed based on clinical judgment. Values confirmed to be physiologically or clinically implausible were treated as missing. Variables with more than 20% missing data were subsequently excluded, and remaining missing values were imputed using multiple imputation methods. Given the relatively balanced distribution between delirium-positive and delirium-negative cases, no additional techniques were applied to address class imbalance.

For feature selection, Spearman correlation coefficients were initially calculated to assess correlations among candidate predictors, and highly correlated variables (absolute correlation coefficient > 0.5) were removed based on both statistical results and clinical relevance. Variance inflation factor (VIF) analysis was then conducted to further detect multicollinearity, and variables with VIF values greater than 5 were excluded. Subsequently, the Boruta algorithm based on random forests was applied for feature selection. This method introduces randomly permuted “shadow features” and compares their importance Z-scores with those of original features across multiple iterations to robustly identify variables significantly associated with the outcome (20). The Boruta algorithm was used to identify all relevant candidate features. Subsequently, to improve model parsimony and clinical applicability, feature subsets were further refined during model development using iterative evaluation within the XGBoost framework, retaining variables that contributed most to predictive performance while minimizing redundancy.

### 2.5 Model development, evaluation, and interpretation

Using a ten-times resampling strategy, the MIMIC-IV dataset was randomly split into a training set (80%) and an internal validation set (20%). To minimize the risk of data leakage, only variables obtained during the initial 24 h after ICU admission were included in model development. Prediction models were developed using four machine learning algorithms: logistic regression (LR), eXtreme Gradient Boosting (XGBoost), Adaptive Boosting (AdaBoost), and Gradient Boosting Decision Tree (GBDT). Model discrimination was evaluated using the area under the receiver operating characteristic curve (AUC). Calibration curves were used to assess agreement between predicted probabilities and observed outcomes, while decision curve analysis (DCA) was conducted to evaluate clinical usefulness. Precision–recall (PR) curves were additionally employed to assess the ability of the models to identify delirium-positive cases across different thresholds. Accuracy, sensitivity, specificity, positive predictive value, negative predictive value, and F1 score were also calculated to comprehensively evaluate model performance. Model hyperparameters were optimized using grid search. SHapley Additive exPlanations (SHAP) were applied to interpret the optimal model, and SHAP summary plots, SHAP importance plots, and SHAP dependence plots were used to illustrate the relative contributions and directional effects of individual features on model predictions.

### 2.6 External validation and development of the prediction tool

To evaluate model generalizability, the eICU-CRD database was used as an independent external validation cohort. The optimal model trained on the MIMIC-IV dataset was directly applied to the external cohort under consistent inclusion criteria, variable definitions, and data preprocessing procedures. Model performance was comprehensively assessed using AUC, accuracy, sensitivity, specificity, positive predictive value, negative predictive value, and F1 score. Based on the stable performance observed in external validation, a prediction tool was developed using the final optimal model to provide intuitive and convenient support for early delirium risk assessment in malnourished elderly ICU patients.

### 2.7 Statistical analysis

Normality of continuous variables was assessed using the Shapiro–Wilk test. Normally distributed variables were expressed as mean ± standard deviation and compared using one-way analysis of variance, whereas non-normally distributed variables were presented as median (interquartile range) and compared using the Kruskal–Wallis test. Categorical variables were expressed as frequencies and percentages and compared using the chi-square test. A two-sided *P* value < 0.05 was considered statistically significant. All statistical analyses were performed using R version 4.2.3 (R Foundation for Statistical Computing, Vienna, Austria) and Python version 3.11.4 (Python Software Foundation, Wilmington, DE, USA).

## 3 Results

### 3.1 Baseline characteristics

**Figure 1:**
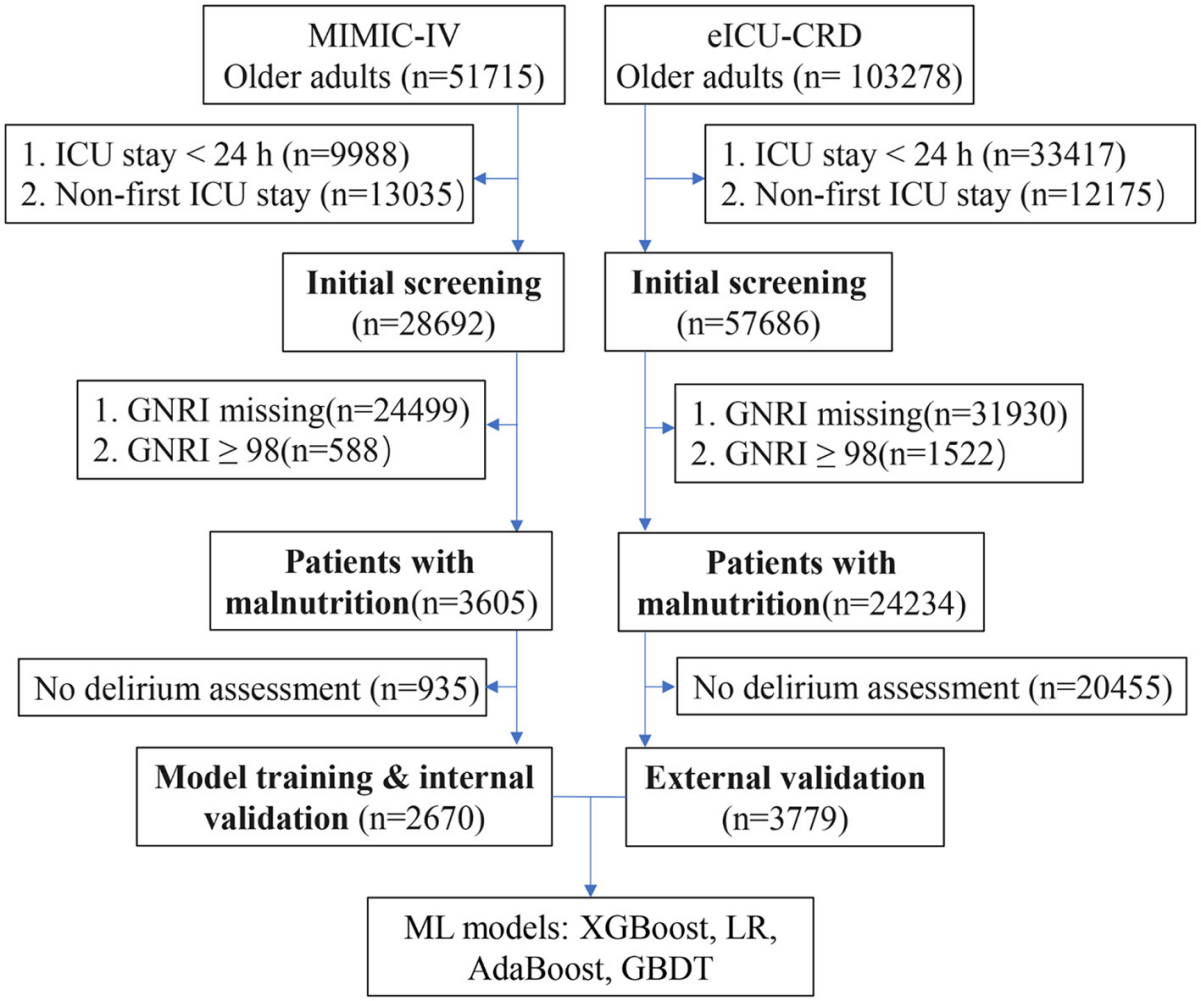
Patient Selection Flowchart**:** the flowchart illustrates the process of patient inclusion and exclusion from the MIMIC-IV and eICU-CRD databases. Elderly patients (≥65 years) with malnutrition (GNRI < 98) were screened according to predefined criteria. Patients with ICU length of stay < 1 day or without delirium assessment records were excluded. Only the first ICU admission during the first hospitalization was included. GNRI, Geriatric Nutritional Risk Index; XGBoost, eXtreme Gradient Boosting; LR, logistic regression; AdaBoost, Adaptive Boosting; GBDT, Gradient Boosting Decision Tree.

According to the predefined inclusion and exclusion criteria, a total of 6,449 malnourished elderly patients were finally included in this study, including 2,670 patients from the MIMIC-IV database and 3,779 patients from the eICU-CRD database. The study flowchart is shown in Figure 1. Baseline characteristics of the included patients are summarized in Table 1. The MIMIC-IV and eICU-CRD cohorts demonstrated good comparability in overall population composition, with similar distributions of age, sex, height, and body weight, as well as comparable ranges and trends in key vital signs, laboratory variables, and treatment-related factors. Significant differences were observed between patients with delirium and those without delirium across multiple clinically relevant variables. In the MIMIC-IV cohort, patients who developed delirium had significantly higher body temperature, heart rate, and respiratory rate, as well as elevated white blood cell count, fasting blood glucose, lactate, and creatinine levels compared with non-delirium patients. In terms of disease severity, the delirium group exhibited significantly higher SOFA (Sequential Organ Failure Assessment) scores [8.0 (5.0–10.0) vs. 4.0 (2.0–7.0)]. Regarding clinical outcomes, the 28-day mortality rate was significantly higher in the delirium group than in the non-delirium group (32.7% vs. 18.1%, *P* = 0.013). Similar findings were observed in the eICU-CRD cohort.

**Table 1:** Baseline characteristics of malnourished elderly ICU patients stratified by delirium status.

Variables	MIMIC-IV			eICU-CRD		
	No delirium (*n* = 1,097)	Delirium (*n* = 1,573)	*P*	No delirium (*n* = 2,645)	Delirium (*n* = 1,134)	*p*
Demographics						
Age (years)	76.0 [70.0; 84.0]	75.0 [70.0; 83.0]	0.026	76.0 [70.0; 83.0]	77.0 [70.0; 84.0]	0.008
Gender, *N* (%)						0.801
Female	529 (48.2%)	688 (43.7%)	0.024	1295 (49.0%)	561 (49.5%)	
Male	568 (51.8%)	885 (56.3%)		1350 (51.0%)	573 (50.5%)	
Weight (kg)	76.4 [64.8; 88.8]	77.5 [64.8; 93.5]	0.031	77.1 [64.6; 91.0]	75.5 [63.5; 91.1]	0.228
Height (cm)	168.0 [160.0; 175.0]	168.0 [160.0; 175.0]	0.281	167.6 [160.0; 175.3]	167.8 [160.2; 177.8]	0.231
Vital signs						
T (°C)	36.8 [36.6; 37.0]	36.9 [36.6; 37.2]	< 0.001	36.7 [36.6; 37.0]	36.8 [36.5; 37.1]	0.033
HR (bpm)	82.0 [72.0; 94.0]	84.0 [74.0; 97.0]	0.003	83.0 [72.0; 95.0]	86.0 [75.0; 97.0]	< 0.001
RR (bpm)	19.0 [17.0; 22.0]	20.0 [17.0; 23.0]	0.002	19.0 [17.0; 22.0]	20.0 [17.0; 23.0]	0.137
SBP (mmHg)	112.0 [102.0; 124.0]	111.0 [103.0; 121.0]	0.055	118.0 [106.0; 134.0]	117.0 [105.0; 131.8]	0.041
DBP (mmHg)	61.0 [55.0; 69.0]	61.0 [56.0; 68.0]	0.493	62.0 [56.0; 70.0]	62.0 [56.0; 70.0]	0.943
MBP (mmHg)	75.0 [69.0; 82.0]	74.0 [69.0; 81.0]	0.605	79.0 [71.0; 89.0]	78.0 [70.0; 88.0]	0.052
SpO_2_ (%)	97.0 [95.0; 98.0]	97.0 [96.0; 99.0]	< 0.001	97.0 [96.0; 98.0]	97.0 [96.0; 99.0]	0.323
Laboratory indicators						
WBC (m/uL)	10.8 [7.8; 15.6]	12.2 [8.5; 17.5]	< 0.001	10.6 [7.7; 14.8]	11.5 [8.7; 16.1]	< 0.001
PLT (K/uL)	183.0 [130.0; 253.0]	181.0 [127.0; 255.0]	0.716	195.0 [143.0; 250.0]	190.5 [142.3; 253.5]	0.589
Hb (g/dL)	10.3 [8.8; 11.8]	10.4 [8.7; 12.1]	0.183	10.7 [9.1; 12.3]	10.6 [9.2; 12.4]	0.946
RDW (%)	14.7 [13.7; 16.3]	14.9 [13.8; 16.6]	0.006	15.1 [14.1; 16.8]	15.1 [14.1; 16.7]	0.543
HCT (%)	31.6 [27.2; 36.1]	32.3 [27.5; 37.5]	0.006	32.7 [28.0; 37.4]	32.5 [28.2; 37.6]	0.907
Alb (g/dL)	3.1 [2.7; 3.4]	2.9 [2.5; 3.3]	< 0.001	2.9 [2.5; 3.3]	2.8 [2.3; 3.2]	< 0.001
Na (mEq/L)	138.0 [135.0; 141.0]	139.0 [135.0; 142.0]	< 0.001	139.0 [136.0; 141.0]	139.7 [136.3; 142.5]	< 0.001
K (mEq/L)	4.2 [3.8; 4.7]	4.2 [3.8; 4.8]	0.123	4.1 [3.8; 4.5]	4.1 [3.8; 4.6]	0.597
Ca (mg/dL)	8.3 [7.8; 8.7]	8.2 [7.6; 8.7]	0.076	8.4 [7.9; 8.8]	8.3 [7.8; 8.8]	0.013
Cl (mEq/L)	104.0 [99.0; 107.0]	104.0 [99.0; 108.0]	0.144	104.0 [100.0; 107.5]	105.0 [100.5; 108.8]	< 0.001
Glu (mg/dL)	130.0 [106.0; 171.0]	141.0 [111.0; 189.0]	< 0.001	130.0 [107.0; 163.0]	134.5 [110.8; 169.0]	0.003
AG (mEq/L)	14.0 [12.0; 17.0]	15.0 [12.0; 18.0]	< 0.001	10.3 [8.0; 14.0]	11.0 [8.5; 15.0]	< 0.001
ALT (IU/L)	25.0 [15.0; 55.0]	27.0 [15.0; 67.0]	0.137	23.0 [15.0; 40.0]	25.6 [16.0; 49.0]	< 0.001
AST (IU/L)	38.0 [22.0; 87.0]	45.0 [24.0; 112.0]	0.003	27.0 [18.5; 53.5]	31.0 [20.0; 70.0]	< 0.001
BUN (mg/dL)	24.0 [15.0; 38.0]	28.0 [18.0; 46.0]	< 0.001	25.0 [16.5; 40.0]	29.0 [18.0; 46.0]	< 0.001
Cr (mg/dL)	1.1 [0.8; 1.6]	1.3 [0.9; 2.1]	< 0.001	1.2 [0.8; 1.8]	1.3 [0.9; 2.1]	0.001
pH	7.4 [7.3; 7.4]	7.3 [7.3; 7.4]	< 0.001	7.4 [7.3; 7.4]	7.4 [7.3; 7.4]	0.230
PO_2_ (mmHg)	89.0 [47.0; 191.0]	78.0 [46.0; 151.0]	0.001	103.0 [79.0; 145.7]	106.0 [81.5; 153.0]	0.052
PCO_2_ (mmHg)	40.0 [35.0; 46.0]	41.0 [35.0; 49.0]	0.001	39.9 [34.0; 47.0]	39.0 [33.3; 47.0]	0.180
Lac (mmol/L)	1.7 [1.2; 2.5]	1.9 [1.3; 3.1]	< 0.001	1.6 [1.1; 2.3]	1.7 [1.1; 2.6]	0.006
PT (sec)	14.3 [12.6; 17.5]	14.6 [12.7; 17.9]	0.229	15.0 [13.1; 18.1]	15.5 [13.4; 18.8]	0.003
APTT (sec)	31.9 [27.9; 41.5]	31.7 [27.7; 40.7]	0.624	33.6 [29.0; 41.7]	34.1 [29.4; 43.9]	0.027
INR (ratio)	1.3 [1.1; 1.6]	1.3 [1.2; 1.6]	0.038	1.3 [1.1; 1.6]	1.3 [1.1; 1.6]	0.011
Comorbidities						
HF, *N* (%)	454 (41.4%)	625 (39.7%)	0.414	454 (17.2%)	138 (12.2%)	< 0.001
AF, *N* (%)	457 (41.7%)	721 (45.8%)	0.036	422 (16.0%)	159 (14.0%)	0.144
Hypertension, N (%)	844 (76.9%)	1246 (79.2%)	0.176	354 (13.4%)	160 (14.1%)	0.586
DM, *N* (%)	133 (12.1%)	154 (9.8%)	0.064	62 (2.3%)	26 (2.3%)	1.000
Hyperlipidemia, *N* (%)	588 (53.6%)	734 (46.7%)	< 0.001	72 (2.7%)	26 (2.3%)	0.516
COPD, *N* (%)	103 (9.4%)	250 (15.9%)	< 0.001	261 (9.9%)	107 (9.4%)	0.726
Pneumonia, *N* (%)	70 (6.4%)	118 (7.5%)	0.300	398 (15.0%)	198 (17.5%)	0.069
CKD, *N* (%)	283 (25.8%)	448 (28.5%)	0.137	191 (7.2%)	63 (5.6%)	0.071
Cirrhosis, *N* (%)	72 (6.6%)	109 (6.9%)	0.770	18 (0.7%)	8 (0.7%)	1.000
Intravenous Infusions						
Dobutamine, *N* (%)	25 (2.3%)	70 (4.5%)	0.004	47 (1.8%)	22 (1.9%)	0.833
Dopamine, *N* (%)	32 (2.9%)	74 (4.7%)	0.026	79 (3.0%)	46 (4.1%)	0.113
Epinephrine, *N* (%)	62 (5.7%)	146 (9.3%)	0.001	37 (1.4%)	24 (2.1%)	0.143
Norepinephrine, *N* (%)	282 (25.7%)	789 (50.2%)	< 0.001	346 (13.1%)	305 (26.9%)	< 0.001
Phenylephrine, *N* (%)	241 (22.0%)	516 (32.8%)	< 0.001	119 (4.5%)	85 (7.5%)	< 0.001
Other indicators						
GNRI	87.9 [81.9; 92.3]	84.9 [78.6; 90.8]	< 0.001	83.4 [75.9; 89.3]	80.4 [73.0; 86.4]	< 0.001
Ventilation, *N* (%)	907 (82.7%)	1457 (92.6%)	< 0.001	630 (23.8%)	486 (42.9%)	< 0.001
Balance_24h (ml)	636 [−895; 2934]	1434 [−576; 4103]	< 0.001	217 (8.2%)	215 (19.0%)	< 0.001
SOFA	4.0 [2.0; 7.0]	8.0 [5.0; 10.0]	< 0.001	4.0 [2.0; 6.0]	6.0 [3.0; 8.0]	< 0.001
GCS	15.0 [14.0; 15.0]	15.0 [13.0; 15.0]	< 0.001	15.0 [13.0; 15.0]	12.0 [8.0; 14.0]	< 0.001
28-day mortality, *N* (%)	199 (18.1%)	514 (32.7%)	< 0.001	217 (8.2%)	215 (19.0%)	< 0.001

T, temperature; HR, heart rate; RR, respiratory rate; SBP, systolic blood pressure; DBP, diastolic blood pressure; MBP, mean blood pressure; SpO_2_, peripheral oxygen saturation; WBC, white blood cell count; PLT, platelet count; Hb, hemoglobin; RDW, red cell distribution width; HCT, hematocrit; Alb, albumin; Na, sodium; K, potassium; Ca, calcium; Cl, chloride; Glu, glucose; AG, anion gap; ALT, alanine aminotransferase; AST, aspartate aminotransferase; BUN, blood urea nitrogen; Cr, creatinine; pH, potential of hydrogen; PO_2_, partial pressure of oxygen; PCO_2_, partial pressure of carbon dioxide; Lac, lactate; PT, prothrombin time; APTT, activated partial thromboplastin time; INR, international normalized ratio; HF, heart failure; AF, atrial fibrillation; DM, diabetes mellitus; COPD, chronic obstructive pulmonary disease; CKD, chronic kidney disease; GNRI, Geriatric Nutritional Risk Index; GCS, Glasgow Coma Scale; SOFA, Sequential Organ Failure Assessment.

### 3.2 Feature selection

**Figure 2:**
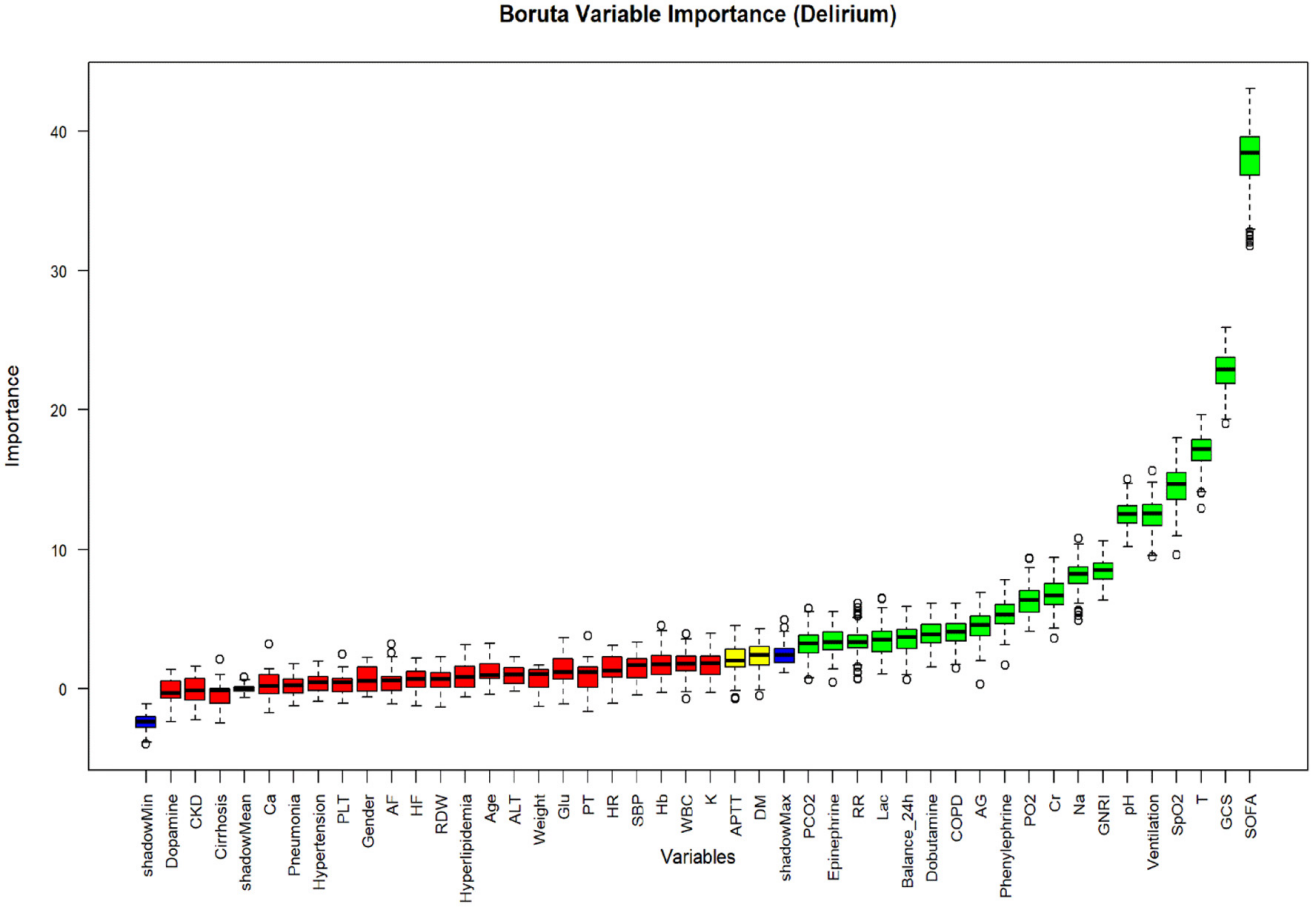
Feature selection analyzed by Boruta algorithm: the Boruta algorithm was applied to identify important predictors associated with delirium. The *x*-axis represents the features, and the y-axis represents the importance Z-scores. Green features represent confirmed important variables, red features indicate unimportant variables that were excluded, yellow features denote tentative importance, and blue features correspond to shadow features used for comparison. Only confirmed important variables were retained for model development.

During the variable selection process, candidate variables were first screened based on missingness, and variables with more than 20% missing data were excluded. Subsequently, correlation heatmaps and variance inflation factor (VIF) analysis were applied to assess collinearity among the remaining variables (Appendix Figure 1), identifying features with strong correlations or multicollinearity. Based on statistical results and clinical interpretability, mean blood pressure (MBP), hematocrit (HCT), albumin, and other redundant variables were further removed. The Boruta feature selection method was then applied to further refine the feature set. This approach determines the relevance of features by comparing the importance of original variables with that of randomly generated “shadow features.” As shown in Figure 2, green features represent confirmed important variables included in the model, red features indicate unimportant variables that were excluded, yellow features denote tentative importance, and blue features correspond to shadow features used for comparison. Ultimately, Boruta identified 19 important features significantly associated with delirium occurrence, including SOFA score, GCS (Glasgow Coma Scale) score, body temperature (T), and peripheral oxygen saturation (SpO_2_).

### 3.3 Model development and performance evaluation

**Figure 3:**
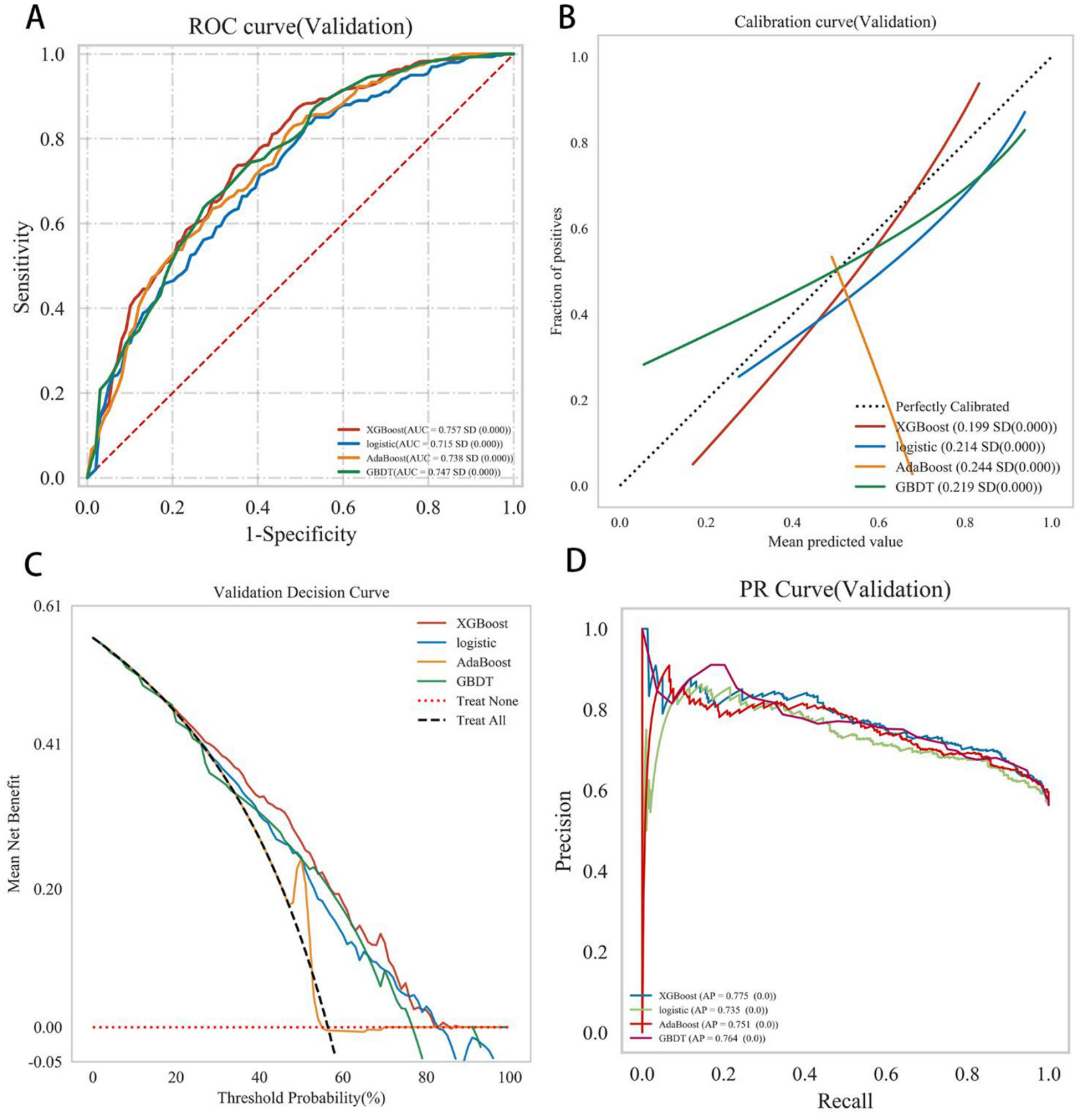
Performance evaluation of machine learning models. **(A)** Receiver operating characteristic (ROC) curves showing the discriminative ability of different models. **(B)** Calibration plots assessing the agreement between predicted probabilities and observed outcomes. **(C)** Decision curve analysis (DCA) evaluating the net clinical benefit across different threshold probabilities. **(D)** Precision–recall (PR) curves illustrating model performance in identifying delirium-positive cases. ROC, receiver operating characteristic; DCA, decision curve analysis; PR, precision–recall; AUC, area under the curve.

Four binary classification machine learning algorithms were used to develop delirium risk prediction models, including LR, XGBoost, AdaBoost, and GBDT. A ten-times resampling strategy was applied to construct training and validation datasets for model performance evaluation. The predictive performance of the models is presented in Figure 3 and Table 2. Receiver operating characteristic (ROC) curve analysis (Figure 3A) demonstrated that the XGBoost model achieved the best discriminative performance in the validation cohort (AUC = 0.757). Calibration curves (Figure 3B) showed that the XGBoost model exhibited the best agreement between predicted probabilities and observed outcomes, with the lowest Brier score (0.199), indicating superior calibration. Decision curve analysis (DCA; Figure 3C) further revealed that the XGBoost model provided the highest net benefit across threshold probabilities ranging from 0% to 90%. Precision–recall (PR) curve analysis (Figure 3D) showed that the XGBoost model achieved the highest average precision (AP = 0.775). In addition, the XGBoost model demonstrated stable performance across other metrics (Table 2), including accuracy (0.665), sensitivity (0.628), specificity (0.712), positive predictive value (0.738), negative predictive value (0.597), and F1 score (0.679). Considering overall discrimination, calibration, clinical utility, and performance stability, the XGBoost model was selected as the optimal prediction model. Using grid search optimization, the final model incorporated seven variables: SOFA score, GCS score, body temperature (T), GNRI, peripheral oxygen saturation (SpO_2_), pH value, and mechanical ventilation.

**Table 2:** Performance evaluation of machine learning models.

Models	AUC	Accuracy	Sensitivity	Specificity	PPV	NPV	F1 Score
Training set							
XGBoost	0.804	0.726	0.708	0.753	0.809	0.636	0.755
Logistic regression (LR)	0.728	0.674	0.680	0.664	0.749	0.585	0.713
AdaBoost	0.765	0.721	0.786	0.625	0.755	0.665	0.770
GBDT	0.776	0.718	0.746	0.677	0.773	0.644	0.759
Validation set							
XGBoost	0.757	0.665	0.628	0.712	0.738	0.597	0.679
Logistic regression (LR)	0.715	0.648	0.668	0.622	0.696	0.592	0.681
AdaBoost	0.738	0.672	0.761	0.558	0.690	0.644	0.724
GBDT	0.747	0.684	0.711	0.648	0.723	0.634	0.717
External validation set							
XGBoost	0.689	0.670	0.540	0.726	0.457	0.786	0.495

XGBoost, extreme gradient boosting; LR, logistic regression; AdaBoost, adaptive boosting; GBDT, gradient boosting decision tree; AUC, area under the receiver operating characteristic curve; PPV, positive predictive value; NPV, negative predictive value; F1 score, harmonic mean of precision and recall.

### 3.4 Model interpretation

**Figure 4:**
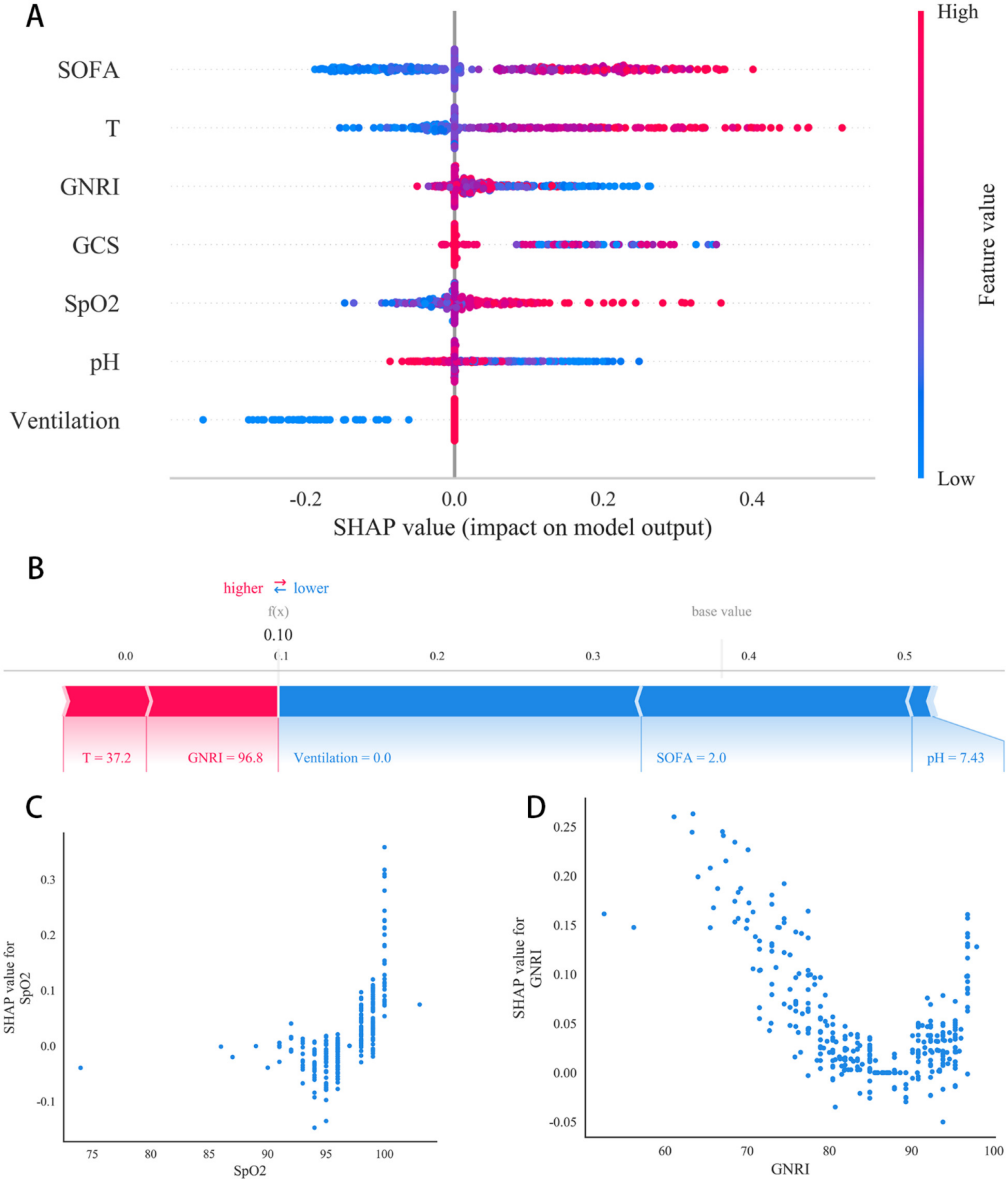
SHAP-based interpretation of the XGBoost model. **(A)** SHAP summary plot showing the overall importance and impact of each feature on model output. The x-axis represents SHAP values, and the y-axis lists the corresponding features. Each point represents an individual patient, with color indicating feature value (red: higher values; blue: lower values). **(B)** SHAP force plot illustrating the contribution of each feature to the predicted delirium risk for an individual patient. Red arrows indicate positive contributions (increased risk), and blue arrows indicate negative contributions (decreased risk). **(C)** SHAP dependence plot for peripheral oxygen saturation (SpO_2_), demonstrating the relationship between SpO_2_ values and their corresponding SHAP values. **(D)** SHAP dependence plot for the Geriatric Nutritional Risk Index (GNRI), showing the nonlinear relationship between GNRI and its contribution to delirium risk. The SHAP dependence plots represent relationships within the malnourished population (GNRI < 98) and should not be extrapolated to patients with normal or high nutritional status. SHAP, SHapley additive explanations; SpO_2_, peripheral oxygen saturation; GNRI, geriatric nutritional risk index.

SHAP (Shapley Additive Explanations), GNRI (Geriatric Nutritional Risk Index) were applied to interpret the predictions of the final model at both global and local levels. Global interpretability was visualized using the SHAP summary plot (Figure 4A), in which the y-axis represents features and the x-axis indicates the magnitude of each feature's contribution to the model output. Each point represents an individual sample, with red and blue colors indicating higher and lower feature values, respectively. The summary plot showed that features with red points predominantly located on the right side included SOFA score, body temperature, and SpO_2_, suggesting that higher values or presence of these features were associated with increased predicted delirium risk. In contrast, blue points for GCS score, GNRI, and pH were mainly distributed on the right side, indicating that lower values of these features were associated with increased delirium risk. Additionally, the absence of mechanical ventilation was associated with a lower predicted risk of delirium. Local interpretability was illustrated using SHAP force plots (Figure 4B) and SHAP dependence plots (Figures 4C , D), which visually demonstrate the direction and magnitude of individual feature contributions to specific predictions. In the SHAP force plot, each Shapley value is represented by an arrow, with red arrows indicating positive contributions (increasing predicted risk) and blue arrows indicating negative contributions (decreasing predicted risk). As shown in Figure 4B, for a representative patient, body temperature of 37.2 °C and a GNRI score of 96.8 increased the predicted delirium risk, whereas the absence of mechanical ventilation, a SOFA score of 2, and a pH value of 7.43 reduced the predicted risk, resulting in a final delirium probability of 10%. SHAP dependence plots demonstrated that the relationship between SpO_2_ and delirium risk was not strictly linear (Figure 4C). Higher SpO_2_ levels were associated with increased SHAP values, indicating a positive contribution to delirium risk, whereas SHAP values in the lower SpO_2_ range were more dispersed without a consistent trend. As shown in Figure 4D, GNRI exhibited a nonlinear relationship with SHAP values, with lower GNRI levels corresponding to higher positive SHAP values, while SHAP values gradually decreased and approached zero as GNRI increased, suggesting a diminished impact at higher GNRI levels.

### 3.5 External validation and development of the prediction tool

**Figure 5:**
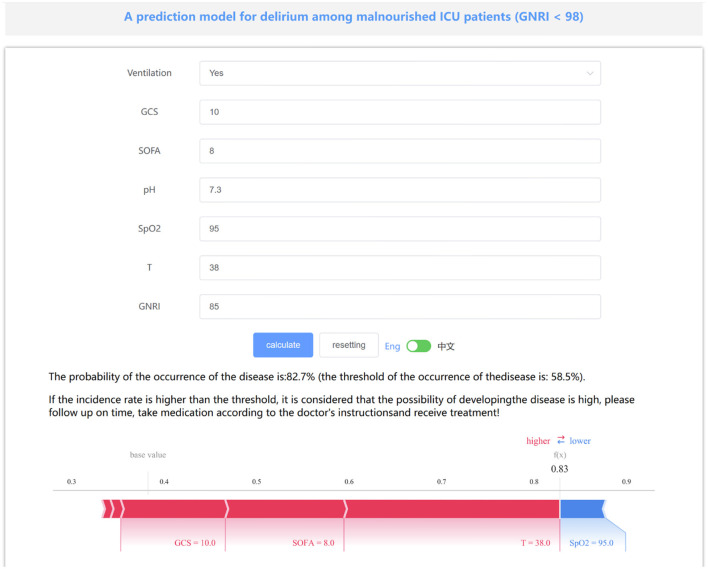
Interface and example output of the delirium risk prediction tool: the figure presents the user interface of the developed prediction tool and an example of its output. Clinical variables, including body temperature, SpO_2_, pH, GNRI, mechanical ventilation status, SOFA score, and GCS score, are entered to estimate the probability of delirium. The model provides an individualized risk prediction along with the contribution of each variable to the final prediction. SOFA, sequential organ failure assessment; GCS, glasgow coma scale; GNRI, geriatric nutritional risk index; SpO_2_, peripheral oxygen saturation.

During external validation, the final XGBoost model was independently validated using the eICU-CRD database. As shown in Table 2, the model achieved an AUC of 0.689 in the external cohort, with an accuracy of 0.670, sensitivity of 0.654, specificity of 0.726, positive predictive value of 0.457, negative predictive value of 0.786, and an F1 score of 0.495, indicating moderate and stable discriminative performance across different data sources. Calibration analysis in the external validation cohort showed acceptable but imperfect agreement between predicted and observed delirium risk, with a tendency toward overestimation at higher predicted probabilities, as illustrated by the calibration curve (Appendix Figure 2). The external validation cohort demonstrated a Brier score of 0.230 (95% CI: 0.202–0.258), indicating acceptable overall calibration performance despite some deviation from ideal calibration at higher predicted probabilities. Based on this model, a prediction tool was further developed, as illustrated in Figure 5. For a hypothetical patient with a body temperature of 38 °C, SpO_2_ of 95%, pH of 7.3, GNRI score of 85, receiving mechanical ventilation, SOFA score of 8, and GCS score of 10, the predicted probability of delirium was 82.7%, with GCS score, SOFA score, and body temperature being the main contributors to increased risk.

## 4 Discussion

This study systematically developed and validated a machine learning–based model for predicting the risk of delirium during hospitalization among malnourished elderly patients using two large multicenter intensive care unit (ICU) databases, MIMIC-IV and eICU-CRD. The results demonstrated that delirium was highly prevalent among malnourished elderly ICU patients and was closely associated with more pronounced physiological disturbances, more severe organ dysfunction, and adverse clinical outcomes. Through systematic comparison of multiple machine learning models, the XGBoost model exhibited superior performance in terms of discrimination, calibration, and net clinical benefit, although a decrease in discriminative performance was observed in the external validation cohort (AUC = 0.689), which may reflect differences in patient populations, data structures, and clinical practice patterns between databases. In addition, variations in delirium assessment and variable distributions may contribute to reduced performance. Further evaluation in diverse clinical settings is warranted. Further integration of SHAP (Shapley Additive Explanations), GNRI (Geriatric Nutritional Risk Index) enabled global- and individual-level interpretability of the model, revealing that disease severity, level of consciousness, nutritional risk, and respiratory function–related indicators played key roles in delirium development. Based on these findings, a prediction tool was developed to provide intuitive quantification of individual delirium risk, offering a potentially translatable tool for early identification and clinical decision support in malnourished elderly ICU patients.

Although CAM-ICU and ICDSC have been widely used and recommended by guidelines as standard tools for delirium assessment in the ICU, several limitations remain in clinical practice. Both instruments rely on bedside assessment by healthcare providers, and their accuracy may be influenced by operator experience and subjective judgment, potentially limiting inter-rater reliability (21). In addition, these tools have relatively low sensitivity for hypoactive delirium or fluctuating symptoms, and their application may be restricted in patients receiving deep sedation or with markedly impaired consciousness, making reliable assessment difficult (19, 22). More importantly, CAM-ICU and ICDSC are primarily designed for the identification of established delirium and lack the ability to prospectively predict delirium risk. Elderly patients are inherently at high risk for delirium in the ICU due to reduced physiological reserve, a high burden of comorbidities, and diminished tolerance to stress. Malnutrition, a highly prevalent yet often underestimated and potentially modifiable condition in older adults, may play an important role in the onset and progression of delirium (10). Previous studies have largely focused on disease severity, sedation strategies, and infection-related factors, with limited systematic evaluation of malnutrition specifically in elderly populations (23, 24). Given that malnutrition may affect central nervous system function through multiple mechanisms, including enhanced inflammatory responses, impaired energy metabolism, and neurotransmitter imbalance (25), focusing on delirium risk among malnourished elderly ICU patients may help identify a more homogeneous high-risk population and facilitate the exploration of modifiable risk factors, thereby enabling early warning and precision management of delirium.

In this study, SOFA (Sequential Organ Failure Assessment) score, GCS (Glasgow Coma Scale) score, body temperature, pH value, and mechanical ventilation were identified as important predictors of delirium, highlighting the critical roles of overall disease severity, disruption of physiological homeostasis, and treatment-related factors in delirium development. Elevated SOFA scores and reduced GCS scores reflect multi-organ dysfunction and impaired consciousness, which may increase delirium risk through altered cerebral perfusion, exacerbated neuroinflammation, and reduced cognitive reserve (23, 26). Although both GCS and delirium assessment tools are related to neurological status, GCS primarily reflects the level of consciousness, whereas delirium is characterized by disturbances in attention, cognition, and fluctuating mental status. Therefore, these measures capture different aspects of brain function. In this study, GCS was measured within the first 24 h after ICU admission and reflects baseline neurological status, rather than the condition at the time of delirium diagnosis. Nevertheless, potential overlap between impaired consciousness and delirium assessment cannot be completely excluded, particularly in critically ill or deeply sedated patients, and part of the predictive contribution of GCS may reflect shared manifestations of neurological dysfunction and delirium risk. Elevated body temperature often indicates infection or systemic inflammation and may impair central nervous system function through inflammatory mediator release and oxidative stress (27). Abnormal pH values reflect metabolic or respiratory imbalance, which may interfere with neuronal excitability and cerebral blood flow regulation (28). Mechanical ventilation is frequently accompanied by sedation, sleep disruption, and altered environmental stimuli, further increasing the likelihood of delirium (29). Collectively, these factors constitute a high-risk clinical context for delirium in malnourished elderly ICU patients. Therefore, the identified contributing factors should be interpreted within the context of malnourished patients and may not be generalizable to patients with normal or high nutritional status.

The role of nutritional status in delirium has received increasing attention in recent years, with multiple studies demonstrating a strong association between malnutrition and increased delirium risk in elderly and critically ill patients (9, 10, 30). By specifically focusing on malnourished elderly ICU patients, this study identified the GNRI as an important predictor of delirium risk, with lower GNRI levels associated with higher predicted delirium risk. It should be noted that the observed nonlinear relationship between GNRI and delirium risk reflects a restricted range of malnutrition (GNRI < 98) and does not represent the full U-shaped association reported in general elderly populations. Therefore, this finding should not be interpreted as suggesting that moderate undernutrition is associated with lower risk than normal nutritional status, but rather that, within malnourished patients, relatively better nutritional status is associated with lower delirium risk. This finding is consistent with previous evidence suggesting that malnutrition may increase susceptibility to delirium by exacerbating inflammatory responses, impairing immune function, disrupting neurotransmitter synthesis, and reducing cognitive reserve (31). Compared with single nutritional indicators, GNRI integrates protein nutritional status and body weight, and may therefore be more suitable for risk stratification in elderly critically ill patients, suggesting that malnutrition is not merely a comorbid condition but may actively contribute to delirium development. Importantly, these findings are derived from a population restricted to malnourished patients (GNRI < 98) and therefore should not be generalized to individuals with normal or high nutritional status.

Notably, this study also found that higher SpO_2_ levels were associated with increased delirium risk in the model. While previous studies have emphasized the detrimental effects of hypoxemia on central nervous system function (32), emerging evidence suggests that excessive oxygen exposure or hyperoxia may also exert adverse neurological effects through oxidative stress, impaired cerebral blood flow regulation, and exacerbation of neuroinflammation (33, 34). In the ICU setting, higher SpO_2_ levels are often accompanied by higher inspired oxygen concentrations or more aggressive respiratory support, and may also reflect greater disease severity or treatment intensity. Therefore, this association should be interpreted with caution, as it may reflect underlying confounding factors such as disease severity, oxygen therapy, or mechanical ventilation, rather than a direct causal relationship. This finding should be considered exploratory and hypothesis-generating, and should not be over-interpreted as evidence of a causal relationship between higher SpO _2_ levels and increased delirium risk. Further investigation is required to clarify the relationship between SpO _2_ and delirium risk.

This study leveraged a large single-center ICU database (MIMIC-IV) and a multicenter ICU database (eICU-CRD) to develop and externally validate a delirium risk prediction model for malnourished elderly patients, enhancing the robustness and generalizability of the findings. By integrating machine learning methods with SHAP-based interpretability analysis, the model enabled precise risk estimation and intuitive identification of key contributing factors, potentially supporting early risk stratification, pending further validation in prospective and real-world clinical settings. However, several limitations should be acknowledged. First, as a retrospective database study, selection bias and unmeasured confounding are unavoidable. In addition, because GNRI calculation required complete albumin, height, and weight data, a substantial proportion of ICU patients were excluded due to missing nutritional variables, which may further limit the representativeness of the study population. Although the model demonstrated moderate discrimination in the external validation cohort, calibration analysis revealed some deviation from the ideal line, suggesting a tendency toward overestimation of delirium risk, particularly at higher predicted probabilities. Second, delirium identification relied on recorded assessment tools and diagnostic codes, which may result in underdiagnosis or misclassification, particularly in patients receiving sedation, mechanical ventilation, or with impaired consciousness; moreover, due to the retrospective nature of the databases, differentiation between prevalent and incident delirium could not be fully ensured, and information on delirium subtypes (e.g., hypoactive vs. hyperactive) was not available, which may further contribute to potential outcome misclassification. Third, certain potentially relevant factors, such as sedative and analgesic use, sleep deprivation, coma status, ICU type, delirium subtypes, dynamic nutritional interventions, and oxygen therapy strategies, were not consistently available in the databases and could not be incorporated into the analysis. Finally, although the model demonstrated moderate and stable predictive performance in external validation, its applicability across different regions, healthcare systems, and prospective clinical settings requires further investigation. This study was not pre-registered, and no head-to-head comparison with existing delirium prediction models or implementation as an online tool was performed, which may limit the completeness of reporting and clinical applicability. In addition, calibration performance was not formally assessed in the external validation cohort, which may limit the comprehensive evaluation of model performance. Furthermore, as this study was restricted to malnourished patients (GNRI < 98), we were unable to evaluate whether the contributing factors for delirium differ across the full spectrum of nutritional status. Future studies including patients with a wider range of GNRI values and stratified analyses are warranted to explore potential heterogeneity in risk factors. Nevertheless, potential overlap between measures of consciousness and delirium assessment cannot be completely excluded and should be interpreted with caution. Although variables were restricted to the early ICU period, patients with greater disease severity may have both prolonged ICU stays and increased delirium risk, and thus residual confounding related to ICU length of stay cannot be completely excluded.

## 5 Conclusion

Using data fromthe MIMIC-IV and eICU-CRD multicenter ICU databases, this study developed and externally validated a machine learning–based model for predicting delirium risk among malnourished elderly ICU patients. The model demonstrated stable discrimination, calibration, and clinical utility, and identified disease severity, level of consciousness, nutritional risk, and respiratory function–related factors as key contributors to delirium development. Combined with SHAP-based interpretability analysis, this approach enables individualized delirium risk assessment and provides valuable support for early identification of high-risk patients and the implementation of targeted prevention and management strategies.

## Data Availability

The datasets analyzed in this study were derived from the publicly available MIMIC-IV and eICU-CRD databases, which require completion of the required training and data use agreements for access. The extracted de-identified dataset supporting the findings of this study has been provided as Supplementary material.
