# Supplementary figures and images for: A machine learning–based prediction model for delirium risk in malnourished elderly ICU patients with SHAP interpretability
Source: Front Nutr. 2026 Jul 20;13:1781117. doi: 10.3389/fnut.2026.1781117 (PMC13430997; doi:10.3389/fnut.2026.1781117)

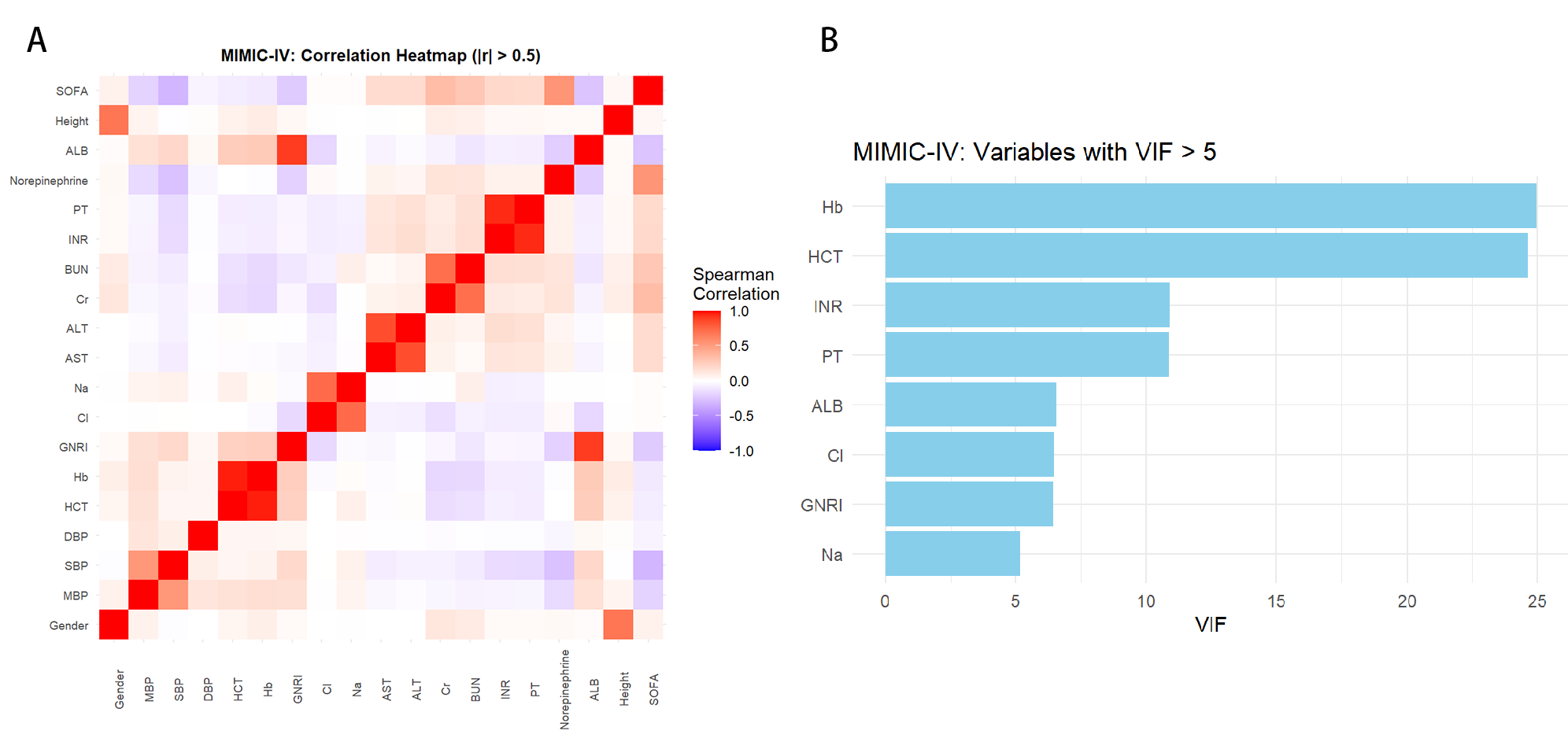

Supplement: Supplementary file 3 [file Image_1.tiff]

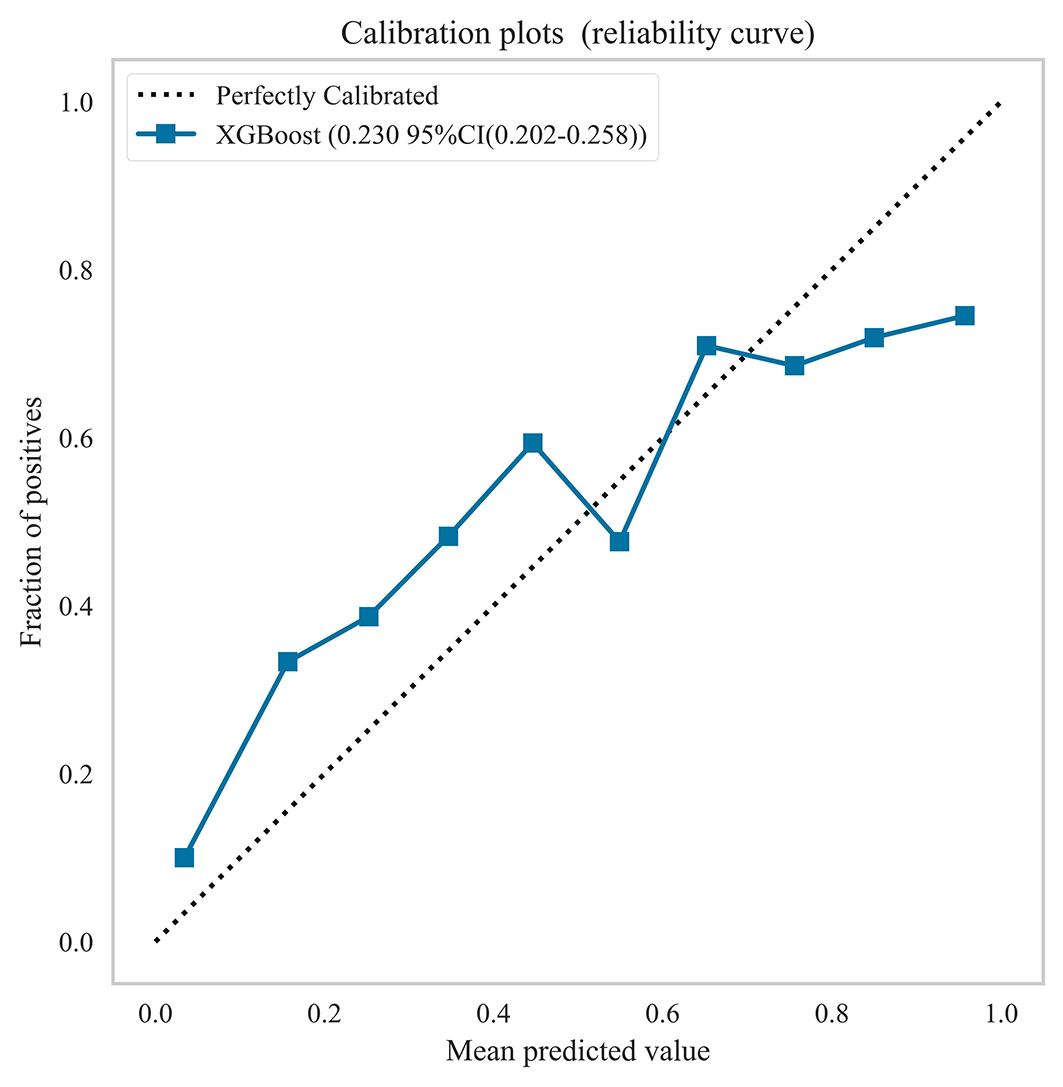

Supplement: Supplementary file 4 [file Image_2.tiff]
